# Two bipolar disorders or one? In reply to commentary by Malhi and Bell

**DOI:** 10.1186/s40345-022-00279-z

**Published:** 2022-12-21

**Authors:** Leonardo Tondo, Alessandro Miola, Marco Pinna, Martina Contu, Ross J. Baldessarini

**Affiliations:** 1 International Consortium for Mood and Psychotic Disorders Research, McLean Hospital, Belmont, MA USA; 2 Department of Psychiatry, Harvard Medical School, Boston, MA USA; 3 Lucio Bini Mood Disorders Center, Via Cavalcanti 28, Cagliari, Italy; 4 Department of Neuroscience, Padova Neuroscience Center, University of Padova, Padua, Italy; 5 Section of Psychiatry, Department of Medical Science and Public Health, University of Cagliari, Cagliari, Italy

Malhi and Bell (2022) provide a critical commentary on the study by Tondo et al. (2022), both published in the *International Journal of Bipolar Disorders*. Their title, *The Bipolar Two-Syndrome Concept: Questioning the Shaping of A Circular Argument for Subtyping a Dimensional Disorder*, makes clear the provocative nature of their commentary. Their major point is that they consider the report by Tondo et al. to be based on subtyping a *dimension* which, by definition, should not be open to categorization.

However, the arguments proposed for this alleged defect are remarkably opinion-based. As the authors have made clear in other recent statements, they do not favor continued existence of bipolar II disorder (BD-2) (Malhi et al. 2020). Instead, they favor a dimensional construct of a single bipolar disorder (BD), in which “without psychotic mania” is a dimensional descriptor. This view, though plausible, is not favored by other experts (Swartz and Suppes 2019; Parker 2021). Malhi and Bell claim that “the creation of bipolar II disorder on arbitrary grounds remains a wholly theoretical construct that lacks any meaningful foundation and clinical utility.” This claim is at variance with clinical reality. Moreover, the dimensional approach to bipolar disorder cannot be considered scientifically or clinically secure or as based other than on description and opinion.

Indeed, management of the even broader concept of manic-depressive illness (MDI) introduced by Emil Kraepelin in the 1890s has been followed by more than a century of contentious debate on ways of dividing this very broad concept into categorical subtypes, including the now nearly universally accepted distinction of bipolar (BD) and major depressive disorder (MDD) subtypes (Trede et al. 2005). In this process, there has been little to guide decisions about recognizing distinguishable sub-syndromes other than clinical description of signs and symptoms, illness-course, and treatment response. More objective measures, such as biological markers or genetic characterization have not been available regarding MDI and BD, nor for most psychiatric disorders.

Contrary to the authors’ claim, the report by Tondo et al. (2022) contains abundant information supporting the descriptive differentiation of two bipolar subtypes, as well as addressing the clinical utility of doing so. Indeed, the discussion section of that report includes the following statement:

We agree that a dimensional model of BD reflects more closely the variability of a complex illness, as was recognized early by Gerald Klerman, but use of a categorical model is more immediate for epidemiological and genomic studies, routine clinical use, scientific communication, and helpful for systematic classifications.

Application of a dimensional concept of BD in clinical settings will eventually call for categorization in some ways. How can one express differences of severity, illness course, treatment response, and outcome if not using some kind of categorization? It is hard to think that a clinician can simply view a patient has having a bit more or less severe BD without considering clinical and liability aspects of such a dimensional approach that avoids considering different forms of the illness. Even the late Hagop Akiskal, who vigorously pursued and expanded on Klerman’s idea of a *bipolar spectrum*, eventually had to invent BD types I, II, II½, III, IV, IV½, V, and VI as categories along a dimensional spectrum between BD and MDD (Akiskal and Pinto 1999).

As a “**fundamental flaw**” Malhi and Bell criticize use of DSM-5 as a diagnostic tool. The “fundamental error” seems to be their disagreement with the currently standard international diagnostic schemes for psychiatric disorders (DSM and ICD), used virtually universally to guide diagnosis for clinical use and for research. That is, their disagreement appears to be with DSM and ICD rather than with a particular research report of observed differences between two clinical syndromes.

Another issue raised by Malhi and Bell is what they consider “**interpretation bias**”. This expression appears to refer to reducing differences between types I and II bipolar disorder syndromes to a “less severe depressive polarity in type II”. That assertion is simply incorrect if referred to Tondo et al. (2022) in which ratings of depression severity yielded higher rating-scale scores for depression in BD-II as well as unsurprisingly lower ratings for [hypo] mania. Moreover, BD-2 compared to BD-1, involved more time depressed, predominant depression overall, a depression-[hypo] mania (DM) course sequence, more rapid cycling involving depressions more than hypomanias, and high risks for suicidal behavior, as well as less time in [hypo] mania and less need for hospitalization. As a consequence, the use of antidepressants is more plausible with BD-2 owing to low risk and low severity of consequences of mood switching from depression into hypomania, whereas antipsychotic medicines or ECT usually are not required.

Malhi and Bell justify their “alternative interpretation” by suggesting that “after more than four decades of research, there is no substantive evidence to support the division of bipolar disorder” along the lines of BD-1 and BD-2, and that circumstance is taken to support the proposal that “BD should not be dichotomized”. Instead, the reality is that research has repeatedly produced results similar to those of the criticized study, as is summarized by Tondo et al. (2022).

In their final remarks, Malhi and Bell claim incorrectly that BD-2 not only is a milder form of BD, but also difficult to differentiate from personality, anxiety, and substance abuse disorders. To some extent, the same confusion can also occur with BD-1. Moreover, based on risks of disability and suicide, BD-2 can hardly be considered a lesser illness than BD-1, even though the two syndromes differ in many ways.

Malhi and Bell conclude that they remain “unconvinced by the findings of this particular study (Tondo et al. 2022), which to our minds supports our dimensional perspective and negates the concept of bipolar II further.” It does seem clear that Malhi and Bell have a different view of BD and that they are not to be persuaded by research data such as that presented by Tondo et al. (2022), finding many substantial differences between BD-2–BD-1.

Moreover, the lack of recognition of a BD-2 can be taken as implying that hypomania cannot exist independently from mania. Hypomania as a distinct syndrome was first described by Berlin psychiatrist Emanuel Ernst Mendel (1839–1907) in his 1881 monograph *Mania* (Shorter 2005):

Patients begin to really enjoy life. Bars, theaters and dances they are now sought out, trips planned and quickly taken…with increased self-confidence they brush aside the doubts about the possible difficulties facing their projects; they also cut off further discussion. In these cases, their egotistical character is particularly striking; they treat the relatives with indifference. Everything is oriented…toward the satisfaction of their own wishes and desires…they pay little attention to money, throwing it out the window, and in short order they run through actually astonishing sums.

Later, Swiss psychoanalyst Carl Gustav Jung (1875–1961) appears first to have described cases which illustrated the diagnostic distinction of manic-depressive illness involving hypomania from that involving mania:

“I would like to publish a number of cases whose peculiarity consists in chronic hypomanic behavior…it is not a question of real mania at all but of a hypomanic state which cannot be regarded as psychotic” (Shorter 2005).

Finally, Malhi and Bell should provide more information on how they would manage the clinical care of BD patients without using any categorical approach. Even an assumption that every patient is unique and requires individual assessment and management does not solve the problem of coordinating syndromal presentations with preferred treatments, of setting clinical standards of care, and teaching of young mental health professionals some guidelines to clinical care and prognosis.

## Data Availability

Not applicable.
